# In Memoriam of John T. Trojanowski, MD, PhD 1946-2022

**DOI:** 10.1186/s13024-022-00530-2

**Published:** 2022-03-24

**Authors:** Hui Zheng, David M. Holtzman

**Affiliations:** 1 Huffington Center on Aging, Baylor College of Medicine, Houston, TX 77030 USA; 2 Department of Neurology, Washington University School of Medicine, St. Louis, MO 63110 USA

John T. Trojanowski, MD, PhD

1946-2022

On February 8, 2022, the world lost a giant in the fields of neurodegenerative disease and brain aging. We lost a beloved colleague and friend in Dr. John Trojanowski. John had a distinguished career most of us could only envy. But he was so much more.

Dr. Trojanowski was a towering figure in the field of neuropathology of neurodegenerative diseases. Following his MD and PhD training at Tufts University and Pathology and Neuropathology Residencies at Massachusetts General Hospital and the Hospital of the University of Pennsylvania, Dr. Trojanowski joined the faculty of the University Pennsylvania in 1981 and stayed there until his passing. Through an academic career that spanned over four decades and together with his life and science partner Dr. Virginia Lee, he built a powerhouse neurodegenerative disease research program and made groundbreaking discoveries that are both wide-ranging and far-reaching. Remarkably, he identified the pathological hallmarks across multiple neurodegenerative diseases: tau and neurofibrillary tangles in Alzheimer’s disease and frontotemporal dementias, Lewy bodies composed of alpha-synuclein that mark Parkinson’s disease and Lewy body dementia, and the TDP43 proteinopathy characteristic of forms of frontotemporal degeneration and amyotrophic lateral sclerosis. Altogether, he co-authored close to 1400 papers and received an astonishing all-time h-index of 247 according to Google Scholar.

John Trojanowski was better known as part of “John and Virginia” than Dr. Trojanowski and his union with Virginia was truly one of a kind and legendary. John’s insights in neuropathology and Virginia’s expertise in biochemistry were a match made in the neurodegeneration research heaven and John’s dark sense of humor and Virginia’s in-your-face (by this we mean in John’s face) persona enlightened every room. People often fondly talk about their public discourse, but the reality is that they could finish each other’s sentences (or e-mails) and John was the ultimate gentleman. Standing at 6 ft 3 in., John was not to be unnoticed but he made sure the spotlight shined on Virginia. The two of them travelled the world together and we had the fortune to get to know them inside conference halls and through social engagements and dinner conversations. It was clear that, like a good bottle of wine that John always enjoyed, their admiration and love for each other only grew with age.

John was a scholar. And his encyclopedic knowledge on the complex pathological and clinical presentations across neurodegenerative diseases were both mesmerizing and intimidating. His penetrating insights brought mixed pathologies and age-related pathological changes to light and humbled our mouse modelers who tend to be fixated on one pathology, one pathway, and one molecule. While trying to understand the role of astrocytes in tau pathology and spreading in mice, one of us (HZ) asked John about the possible disease relevance. He gave me a lecture about the astroglial tau inclusions in various tauopathy conditions and introduced me to a phenomenon termed aging-related tau astrogliopathy (ARTAG). This was followed by e-mail attachments of a dozen of papers. (I have to confess that I did not read all of them.)

John was as energetic as he was knowledgeable. He often responded to e-mails within minutes, regardless of the time. In the beginning, I thought it was bounced back e-mails. Later on, this almost became expected. Indeed, it seemed that John was always working.

Above all, John was extremely generous and collaborative. He provided both of us large number of well-annotated, high-quality, human brain samples that made our work possible [1–5]. We asked for 1 g per sample and was delighted to learn that he has sent us more than 5 g each! When it comes to co-authorship, I quote an e-mail from John that speaks of his generosity and character: “We appreciate very much that you included us in this important study. We are eager for future collaborations with you and your team when the occasion arises … we just wanted to be sure it is appropriate in your mind for us to be listed as collaborators.” What a gentleman!

John was a pioneering neuropathologist, an inspiring leader, a skilled mentor, a devoted husband to Virginia, a beloved colleague and friend to so many, and a kind and generous human being. He will be sorely missed.
